# Multianalytical and Theoretical Tendency for the Clarification of the Binding of Lansoprazole and Albumin Protein

**DOI:** 10.1021/acsomega.5c09146

**Published:** 2026-03-31

**Authors:** Murat Çelik, Cigdem Kanbes-Dindar, Ruqia Khan, Fazal Rehman, Gulsum Ece Meraki, Saharuddin Bin Mohamad, Ali Onur Asap, Fatih Inci, Bengi Uslu

**Affiliations:** † Faculty of Pharmacy, Department of Analytical Chemistry, Ankara University, Ankara 06560, Turkiye; ‡ Department of Chemistry, Quaid-I Azam University Islamabad, Islamabad 45320, Pakistan; § Department of Chemistry, Govt. Girls Degree College Chitti Dheri Mansehra, Hazara University, Dhodial 21300, Pakistan; ∥ Biochemistry Program, Institute of Biological Sciences, Faculty of Science, University Malaya, Kuala Lumpur 50603, Malaysia; ⊥ The Graduate School of Health Sciences, Ankara University, Ankara 06110, Turkiye; # Faculty of Science, Bioinformatics Programme, Institute of Biological Sciences, University of Malaya, Kuala Lumpur 50603, Malaysia; ¶ Centre of Research for Computational Sciences and Informatics for Biology, Bioindustry, Environment, Agriculture and Healthcare, University of Malaya, Kuala Lumpur 50603, Malaysia; ∇ UNAM—National Nanotechnology Research Center, Bilkent University, Ankara 06800, Turkiye; ○ Institute of Materials Science and Nanotechnology, Bilkent University, Ankara 06800, Turkiye

## Abstract

Understanding the interaction between pharmaceutical agents and serum albumin is critical for assessing drug bioavailability, distribution, and pharmacokinetics. In this study, the binding mechanism of lansoprazole (LAN) to bovine serum albumin (BSA) was systematically investigated using spectroscopic, electrochemical, and computational approaches. Fluorescence quenching experiments showed that the interaction follows a dynamic quenching mechanism and a moderate affinity, revealing a binding constant of 1.25, 2.76, and 5.24 × 10^4^ M^–1^ for 287, 298, and 307 K, respectively. Thermodynamic analysis revealed that the binding process is predominantly driven by hydrophobic interactions, as supported by positive enthalpy (Δ*H*) and entropy (Δ*S*) values. UV–vis absorption spectroscopy confirmed structural changes in BSA upon LAN binding, while three-dimensional fluorescence spectroscopy showed changes in the microenvironment of tryptophan and tyrosine residues. Electrochemical studies also supported the formation of a stable, nonelectroactive LAN–BSA complex, leading to decreased peak currents. Molecular docking and molecular dynamics simulations provided atomic-level information, showing that LAN preferentially binds to the I site of BSA, stabilized by hydrogen bonding and hydrophobic interactions. Circular dichroism spectral results indicated a secondary structural change in HSA upon LAN binding. The findings of this study contribute to a better understanding of the pharmacokinetic behavior of LAN, providing valuable information about its therapeutic efficacy and potential drug–protein interactions.

## Graphical abstract

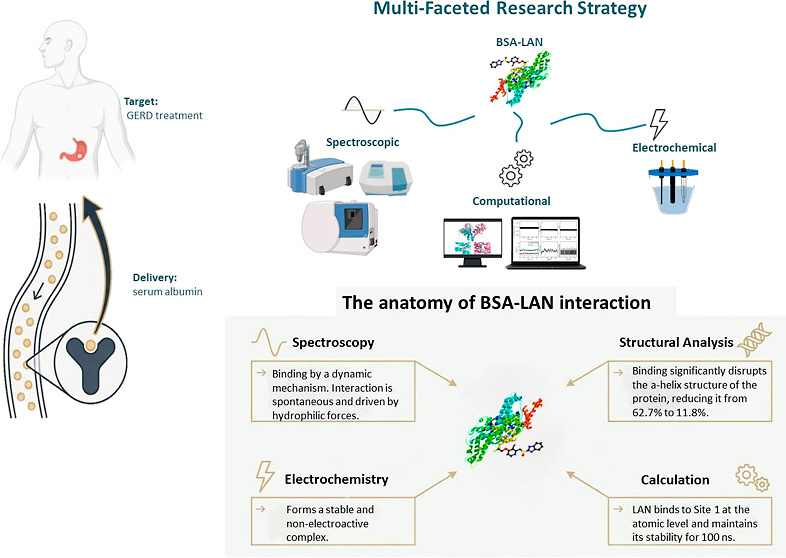

## 1 Introduction

Gastroesophageal reflux disease (GERD) is a chronic condition that is prevalent in the general population. This condition is characterized by the reflux of gastric contents back into the esophagus, which can result in damage to the esophageal mucosa over time. GERD has a markedly detrimental impact on the quality of life of patients, with the potential to precipitate serious complications such as esophageal cancer in the absence of appropriate intervention over an extended period.^1,2^ Proton pump inhibitors, such as FDA-approved lansoprazole (LAN), are a class of pharmaceutical agents commonly used to treat this disease.^3^ LAN is a proton pump inhibitor that specifically inhibits gastric acid production, thereby relieving the symptoms caused by acidic reflux and promoting healing of the esophageal mucosa (Figure 1). By reducing acid secretion, this medicine raises the pH of the stomach and thus lowers the acidity of the stomach contents, thereby controlling the symptoms associated with acid reflux. Effective use of LAN improves patients’ quality of life in the management of GERD and can prevent esophageal damage.^4–6^

**Figure 1:**
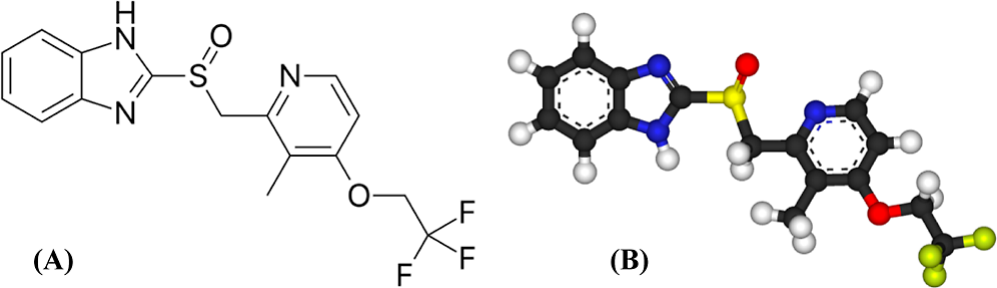
Chemical structure of LAN in 2-D (A) and 3-D (B) formats.

The study of drug–protein binding is of great importance for the elucidation of the effects of drugs on the body and the optimization of their efficacy. The transportation of drugs is dependent upon their binding to proteins within the bloodstream, particularly albumin.^7,8^ The extent of this binding determines the distribution, efficacy, half-life, and access to target tissues of the drug. The strength of the drug–protein binding interaction determines the circulation time and duration of the pharmacological effect of the drug. In the case of weak binding, the drug is rapidly eliminated from the body. This factor has a direct impact on the pharmacokinetic and pharmacodynamic profiles of drugs, and it is a crucial parameter that should be taken into account in order to enhance the efficacy of the drug and mitigate its adverse effects in the treatment processes. Moreover, drug–protein interactions may also increase the potential for different drugs to interact with each other, necessitating careful dosage adjustment in multidrug use.^9–11^ In conclusion, a comprehensive investigation of drug–protein interactions is instrumental in the advancement of safer and more efficacious pharmaceuticals as well as in the domain of personalized medicine.

Bovine serum albumin (BSA) is a widely utilized model protein for investigating drug–protein interactions. BSA exhibits structural and functional similarities to human serum albumin, rendering it an optimal model for pharmacokinetic and pharmacodynamic investigations.^10,12,13^ The transportation of drugs in the bloodstream is typically facilitated by their binding to plasma proteins, particularly albumin, which plays a key role in maintaining pharmacokinetic balance and drug bioavailability.^14,15^ This binding process plays a pivotal role in determining the bioavailability, distribution, metabolism, and elimination of drugs.^16–18^ The high binding capacity of BSA allows for a detailed study of drug binding sites, binding kinetics, and thermodynamic properties. Interaction studies performed with this protein provide crucial information for predicting drug efficacy and safety, as well as potential side effects and drug–drug interactions.^12,19,20^ BSA-based analyses offer essential data on drug efficacy and safety, particularly in the context of new drug development. Furthermore, the economic advantage of BSA increases its preference in scientific research.^21^

Recent research has demonstrated the binding mechanism of LAN to BSA.^22^ However, this study’s data has inconsistencies. The LAN interaction with BSA was analyzed using the fluorescence technique, and Förster’s theory of binding distance was found to be 0.19 Å. To confirm the formation of a complex between the drug and BSA, the result must range from 0.5 to 2.0 Å.^23^ The existence of such a number indicates that the data derived from the analysis is untrustworthy. Consequently, to prevent potential drug–drug and drug–food interactions associated with this often-used LAN, the transport mechanism must be accurately elucidated. Furthermore, we used UV correction in the fluorescence study and validated our findings electrochemically. Moreover, while the drug’s binding site to BSA was identified as side 1 with an efficient static mechanism, our research findings indicated that the binding site was really side 1, where a dynamic mechanism proved successful. In another study in the literature, Dombi and colleagues investigated the binding properties of lansoprazole enantiomers with α_1_-acid glycoprotein (AGP) and human serum albumin (HSA).^24^ In this study, it was reported that the R-enantiomer exhibited a higher affinity for AGP (Δ*G* ≈ −0.4 kcal·mol^–1^) and showed stereoselective binding on HSA. The study evaluated only LAN enantiomers, used HSA as the protein model, and applied high-performance liquid chromatography, fluorescence spectroscopy, and molecular binding methods. In these respects, the study differs fundamentally from ours in terms of both the protein model and the methodological approach. In our study, the commercially available racemic LAN form was investigated, BSA was used as the model protein, and internal filter effect-corrected fluorescence decay analysis, differential pulse voltammetry (DPV), circular dichroism (CD) spectroscopy, and molecular dynamics simulations were used together to comprehensively evaluate binding thermodynamics, static/dynamic decay mechanisms, and protein secondary structure changes. Thus, the objective of this study is to investigate the molecular interaction between LAN, a pharmaceutical agent that has been approved by the Food and Drug Administration (FDA), and BSA. The investigation of drugs is frequently conducted in vitro with the objective of gaining insights into their molecular interaction mechanisms during clinical use. The interaction between lansoprazole and serum albumin plays a crucial role in determining its bioavailability, stability, and transport behavior in the bloodstream. A detailed understanding of this interaction through spectroscopic and theoretical approaches is essential to reveal the molecular forces and binding patterns that govern the pharmacokinetic profile of lansoprazole. This research examines the binding affinity of LAN to BSA, the quenching process, and the associated intermolecular interactions. A variety of spectroscopic and voltammetric techniques were employed, complemented by computational molecular docking and simulation tools. The objective was to characterize the molecular combination of LAN with BSA and assess the impact of this combination on protein conformation. The findings reveal that LAN induces structural changes in BSA upon binding. This study is expected to contribute valuable insights into drug binding and efficacy, potentially benefiting drug design and development. This article presents a comprehensive analysis of the binding interaction between LAN and BSA, integrating experimental and theoretical approaches.

## 2 Materials and Methods

### 2.1 Reagents

Pure BSA (free from globulin and essential fatty acid; purity 99%) and LAN (purity 99%) were supplied by Sigma-Aldrich Co. Also, other chemicals used in the experiments were of analytical grade standards. Merck Millipore was the source of Milli-Q water in this study.

### 2.2 Stock Solutions

The concentration of phosphate buffer solution (PBS) used in the preparation of BSA solution was adjusted to 60 mM and the pH to 7.4. The BSA concentration was estimated (ε_m_ = 36,500 M^–1^ cm^–1^ at 280 nm) from the maximum absorbance value.^25^ A stock solution of LAN was made after its dissolution in dimethyl sulfoxide. It was diluted to the necessary concentration by using PBS with a pH of 7.4.

### 2.3 UV–Vis Absorption Spectral Measurements

Absorption spectra were acquired spectrophotometrically at 298 K using a quartz cuvette with a path length of 1 cm. Additionally, for the correction of the inner filter effect of the observed fluorescence data, absorption spectra (λ = 280–450 nm) of 3 μM BSA at increasing LAN concentrations (0–80 μM with 10 μM increments) were recorded. The absorption spectra of 10 μM BSA without and with 10, 20, 30, 40, and 50 μM LAN concentrations were scanned in the λ range of 220–350 nm. Absorption spectra (λ = 220–350 nm) of 15, 30, 45, and 60 μM LAN were also recorded.

### 2.4 Fluorescence Spectral Measurements

All fluorescence spectra were obtained with a spectrofluorometer using a 10 mm path length cuvette. To maintain the instrumental temperatures at 287, 298, and 307 K for fluorescence experiments, the Peltier-type single-cell thermostat system was employed. In titrations, fluorescence signals (λ_ex_ = 280 nm, λ_em_ = 300–450 nm) of 3 μM BSA were obtained at 287, 298, and 307 K upon inclusion of LAN concentrations (0–80 μM). To ensure adequate interaction between LAN and BSA, the prepared solutions were left for 30 min before measuring the fluorescence spectra.

The 3-D fluorescence signals of 3 μM BSA, 40:3, and 80:3 [LAN]/[BSA] solutions were recorded using λ_ex_ = 220–350 nm (5 nm data pitch) and λ_em_ = 220–500 nm (1 nm data pitch). The synchronous fluorescence signals (λ_em_ = 245–345 nm) of BSA (3 μM) with added concentrations of LAN (0–80 μM with 10 μM intervals) were recorded employing the λ_ex_ and λ_em_ intervals (Δλ) of 15 and 60 nm, respectively.

### 2.5 Electrochemical Measurements

Electrochemical tests were conducted using an AUTOLAB-PGSTAT204 potentiostat/galvanostat (Ecochemie, Netherlands) system to verify the development of a complex between LAN and BSA. A glassy carbon electrode (GCE, diameter: 3 mm) served as the working electrode; an Ag/AgCl electrode functioned as the reference electrode; and a platinum wire acted as the counter electrode in a three-electrode system employing DPV methodology. The GCE surface was cleansed with an alumina slurry and ultrapure water prior to each measurement, all conducted at ambient temperature. The differential pulse voltammograms were obtained for the quantitative analysis of BSA on LAN utilizing 50 μM LAN, with BSA concentrations ranging from 0 to 12 μM.

### 2.6 Circular Dichroism (CD) Measurements

CD spectra of BSA and its 1:1 complex with ligand (BSA/LAN) were recorded by using a JASCO J-815 spectropolarimeter equipped with a Peltier temperature control unit. Measurements were performed at 25 °C under a nitrogen atmosphere in a 1 mm path-length quartz cuvette. Samples were prepared in phosphate-buffered saline (PBS, pH 7.4) with a BSA concentration of 3 μM and a 1:1 molar ratio of BSA to the LAN ligand. Each spectrum was averaged from three consecutive scans. Spectra were collected in the far-UV range (200–250 nm) with a 1 nm data pitch, 2 nm bandwidth, and a scanning speed of 50 nm/min. The data integration time was 4 ms with standard sensitivity and automatic PMT voltage adjustment. Baseline correction and smoothing were performed using Spectra Analysis 2 software. The corrected spectra were analyzed in Microsoft Excel, converting ellipticity (θ, in millidegrees, mdeg) to mean residue ellipticity (MRE, deg·cm^2^·dmol^–1^) for normalization.

#### 2.6.1 Mean Residue Ellipticity Calculation

Ellipticity data in mdeg were converted to Mean Residue Ellipticity (MRE, deg·cm^2^·dmol^–1^) using the equation

$${\left[\theta \right]}_{MRE}=\frac{\theta_{obs}}{10\times c\times n\times l}$$

where [θ]_MRE_ is mean residue ellipticity (deg·cm^2^·dmol^–1^), θ_obs_ is observed ellipticity (mdeg), MRW is mean residue weight (approximately 110 Da for BSA), *c* is protein concentration (mg/mL), and *l* is path length (cm).

#### 2.6.2 Calculation of α-Helix Content

The α-helix content of a protein is commonly estimated from circular dichroism (CD) spectra using the mean residue ellipticity (MRE) value at 208 nm, a wavelength at which α-helical secondary structures show a characteristic negative band.

Calculation formula (Bowman’s equation)

$$\alpha ‐helix\;\left(\%\right)=100\times \left(\frac{-\left[\theta \right]208-4000}{33,000-4000}\right)$$

The equation estimates what fraction of the protein adopts an α-helical structure based on how close the measured MRE_(208)_ is to the theoretical maximum (−33,000). The result is given as a percentage (%), representing the proportion of the polypeptide chain in an α-helix conformation.

- [θ]_208_ = mean residue ellipticity at 208 nm (deg·cm^2^·dmol^–1^)
- 4000 = baseline value for random coil
- 33,000 = reference value for a 100% α-helical structure

### 2.7 FTIR Measurements

Fourier transform infrared (FTIR) spectra were recorded on a Bruker Tensor 27 spectrometer equipped with an attenuated total reflectance (ATR) diamond crystal accessory to investigate the conformational changes of BSA upon interaction with LAN. The spectra were collected in the 4000–600 cm^–1^ range at a spectral resolution of 4 cm^–1^, averaging 32 scans for each sample to improve the signal-to-noise ratio. For each measurement, a small drop of the sample solution was carefully placed on the ATR crystal, ensuring a uniform contact with the surface. The background spectrum was recorded under identical conditions and automatically subtracted from the sample spectra. Also, FTIR spectra of 3 μM BSA and a 12:1 LAN/HSA mixture were scanned in the wavenumber range of 1000–4000 cm^–1^.

### 2.8 Molecular Docking Approach

A computational method was built to verify the optimal binding position of LAN on BSA. Independent docking simulations were conducted after evaluating the two BSA binding locations, remarkably at Sites I and II, using AutoDock 4.2 and AutoDockTools 1.5.7.^26,27^ The protein’s 3-D structure (PDB ID: 4F5S) was disclosed by the Protein Data Bank (PDB), with the lowest resolution enabled at 2.47 Å. The LAN 3D structures were generated using the software Chemsketch, which was subsequently compressed by employing a field of force (MMFF94) upon Avogadro 1.1.1.^28^ The grid box was constructed in the location of Sites I and II to evaluate the binding sites on BSA independently. The cells were 70 × 70 × 70 units in size, and the spacing between the cells was 0.375 Å. The coordinates for Site I are *x* = −4.76, *y* = 30.77, and *z* = 101.15, while the coordinates for Site II are *x* = 10.86, *y* = 17.43, and *z* = 120.61. To identify the optimal binding affinity of LAN on BSA, 100 runs were executed with 150 populations utilizing a Lamarckian genetic algorithm. LigPlot+^29^ and Chimera 1.17.3 tools^30^ were further employed to conduct additional research on the illustration of intermolecular associations.

### 2.9 Molecular Dynamics Simulation Technique

To examine the sustainability of LAN at Site I of BSA, GROMACS 2022 was applied for molecular dynamics (MD) simulation.^31^ By using a Vrescale thermostat^32^ and a Parrinello–Rahman barostat,^33^ the temperature of the program was kept at 300 K, and its pressure was maintained at 1 bar during the 100-simulation production run. First, the LAN topology was generated by applying SwissParam,^33^ subsequently linked with BSA topology to further diminish energy by the steepest descent by utilizing the force field of CHARMM36,^34^ followed by dissolution and eradication with the water method of TIP3P.^35^ The Verlet approach and the particle-mesh Ewald system were employed to figure out the program’s closest neighbor analyses and distant electrostatic relations, respectively.^36^ For evaluation purposes, a trajectory is secured and protected at each 10 ps throughout the simulation, resulting in 5001 rotations that comprise the initial structure.

## 3 Results and Discussion

### 3.1 Spectrofluorometric Determination of the LAN–BSA Interaction

Absolute concentrations inside human bodies are often ambiguous, making total binding to proteins a significant factor in pharmacological research.^37–41^ Fluorescent spectroscopy has shown significant benefit in drug–protein binding investigations due to its high sensitivity, ease of operation, efficiency, and capability for real-time in situ observations.^42,43^ This technique enables elucidation of the LAN-protein binding mechanism and identification of the binding sites. The protein displays inherent fluorescence emission features due to the presence of tryptophan (Trp), phenylalanine (Phe), and tyrosine (Try).^44–49^ However, the fluorescence effect of Phe is restricted. BSA fluorescence is influenced by both Trp and Try at an excitation wavelength of 280 nm.^18^ This study investigated the fluorescence emission signals of BSA at increasing LAN concentrations and in the absence of LAN, using an excitation wavelength of 280 nm. No fluorescence signal was detected for the LAN in the examined emission area. At all temperatures (287, 298, and 307 K), the BSA fluorescence signal progressively diminished with the increase of LAN concentrations. The inner filter effect in fluorescence spectra denotes the reduction of the produced fluorescence signal caused by the absorption of both the excitation light and the emitted light by the sample or other constituents inside the sample.^50,51^ This phenomenon may result in mistakes in fluorescence intensity measurements, particularly when analyzing dense samples or certain optical configurations.^52,53^ The following inner filter effect formula (eq 1) was used to rectify inaccurate data in characterizing the interaction between the LAN and BSA.

1 $$F_{cor}=F_{obs}10^{\left(A_{ex}+A_{em}\right)/2}$$

where *A*_ex_ and *A*_em_ denote the absorbance differences at λ_ex_ and λ_em_, respectively, whereas *F*_obs_ and *F*_cor_ indicate the observed and corrected fluorescence intensities, respectively.^54^ Corrected fluorescence (*F*_cor_) measurements were used in all calculations based on fluorescence data. The corrected fluorescence response of BSA at varying LAN concentrations is shown in Figure 2A–C. At three distinct temperatures (287, 298, and 307 K), the fluorescence signal of BSA diminished by almost 75% as the concentration of LAN rose. BSA fluorescence intensity may vary according to Trp microenvironment changes.^55^ Thus, the BSA-LAN complex is postulated. Increasing the LAN concentration at all temperatures resulted in a progressive quenching of BSA fluorescence and a blue shift in the emission maxima (Figure 2A–C). The polarity of the surrounding fluorophore molecules often influences emission maxima.^56^ The blue shift indicates that amino acid residues have relocated to a more hydrophobic region, influencing the microenvironment of the molecule’s fluorescent segment.^57^

**Figure 2:**
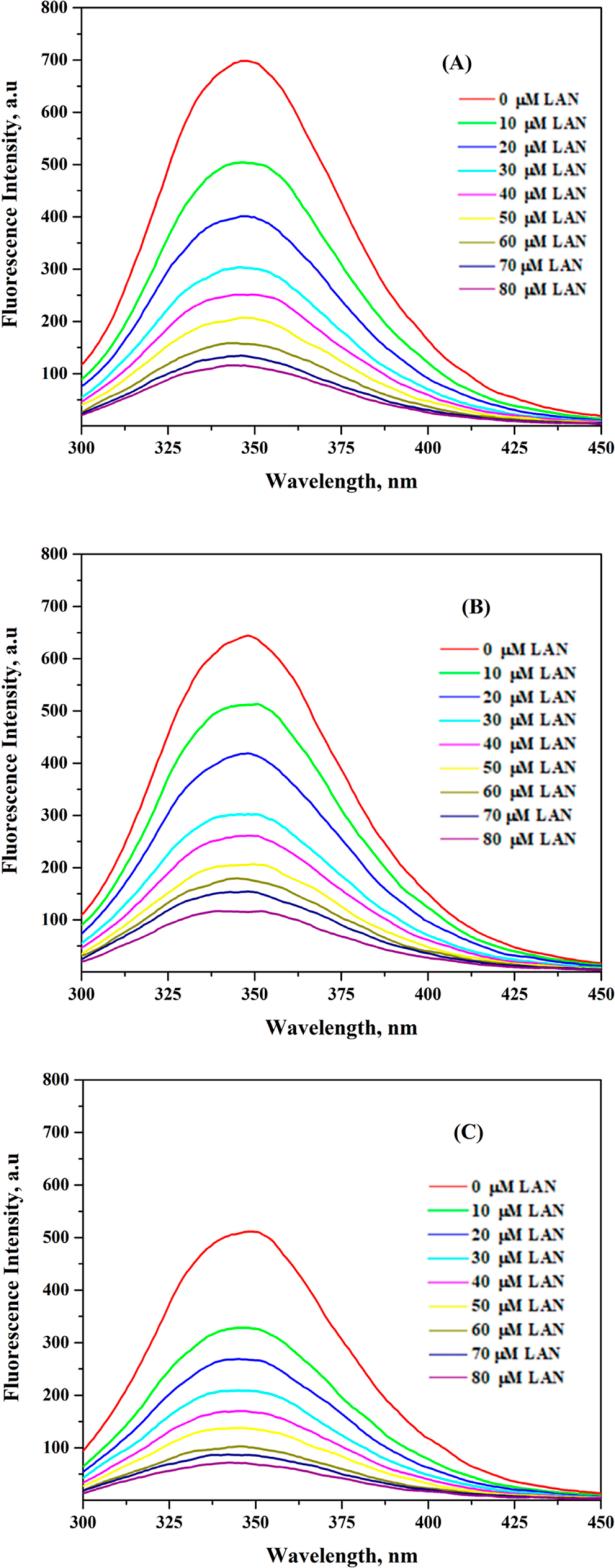
Fluorescence quenching results of BSA with advancing LAN concentrations (spectra “top to bottom”) at (A) 287 K, (B) 298 K, and (C) 307 K. (λ_ex_ = 280 nm; [BSA] = 3 μM; [LAN] = (0–80) μM with 10 μM regular intervals; buffer = PBS 7.4.).

Stern–Volmer plots are created at various temperatures, and then the Stern–Volmer quenching constant (*K*_SV_) for each temperature is obtained from the slope of these plots^56^

2 $$F_{0}/F=K_{SV}\left[Q\right]+1=k_{q}\tau_{0}\left[Q\right]+1$$

The fluorescence intensities without LAN are designated as *F*_0_, whereas those with LAN are designated as *F*. The LAN concentration, *k*_q_, the bimolecular quenching rate constant, and the mean lifetime of the protein without the quencher are denoted by *Q*, τ_0_, and τ, respectively.^58^

The τ_0_ value of BSA was assumed to be 10^–8^, allowing for the calculation of *k*_q_ using the equation below.^59^

3 $$k_{q}=K_{SV}/\tau_{0}$$

Figure 3 exhibits Stern–Volmer plots of LAN–BSA fluorescence at 287, 298, and 307 K. The plots showed strong linearity with a correlation coefficient (*r*) of ≥0.994. *K*_SV_ values from the slope values of various plots are presented in Table 1. The temperature-dependent behavior of *K*_SV_ defines dynamic and static quenching processes.^60^ Increased temperature accelerates molecule diffusion, increasing collisional quenching, whereas weakly bound complex dissociation may reduce static quenching.^61^ Higher temperature brings about diffusion and collision between the ligand and protein molecules, increasing the *K*_SV_ in dynamic quenching.^39,62,63^ Additionally, elevated temperatures facilitate the diffusion and collision between ligands and protein molecules, thereby increasing the *K*_SV_ in dynamic quenching.^55,64^ As the temperature increases for the dynamic mechanism, the *K*_SV_ increases, as stated by Lakowicz.^65^ Although fluorescence lifetime measurements would provide additional confirmation, the temperature-dependent increase in *K*_sv_ values clearly indicates a dynamic quenching mechanism between BSA and the LAN. According to our findings, dynamic quenching seemed to be the mechanism by which BSA and LAN interacted. The *k*_q_ values that were found corroborate our earlier observations that the quenching manner was dynamic. Therefore, the obtained data indicate a dynamic quenching mechanism involving the formation of a stable complex between LAN and BSA. According to the data we obtained, this strongly supports that the interaction between LAN and BSA occurs through a dynamic quenching mechanism. The consistent increase in *K*_sv_ values (from 1.25 × 10^4^ M^–1^ to 7.73 × 10^4^ M^–1^) with increasing temperature (from 287 to 307 K) reflects an increase in diffusion-controlled collisions between LAN and BSA, thereby reflecting the characteristic feature of dynamic quenching additionally, the calculated *K*_q_ values (e.g., 3.62 × 10^12^ M^–1^ s^–1^ at 298 K) reinforce the high efficiency and dynamic nature of this interaction. Thermodynamic analyses also support this interpretation; the positive enthalpy change (Δ*H* = +52.63 kJ mol^–1^) and positive entropy change (Δ*S* = +261.73 J mol^–1^ K^–1^) indicate that the interaction is entropy-driven and that hydrophobic forces play an important role, which is consistent with dynamic complex formation. The negative Gibbs free energy (Δ*G*) values observed at all temperatures (from −22.49 to −27.73 kJ mol^–1^) confirm that the LAN–BSA interaction is a spontaneous process. These findings indicate the presence of a dynamic quenching mechanism involving the formation of a stable complex between LAN and BSA.

**Figure 3:**
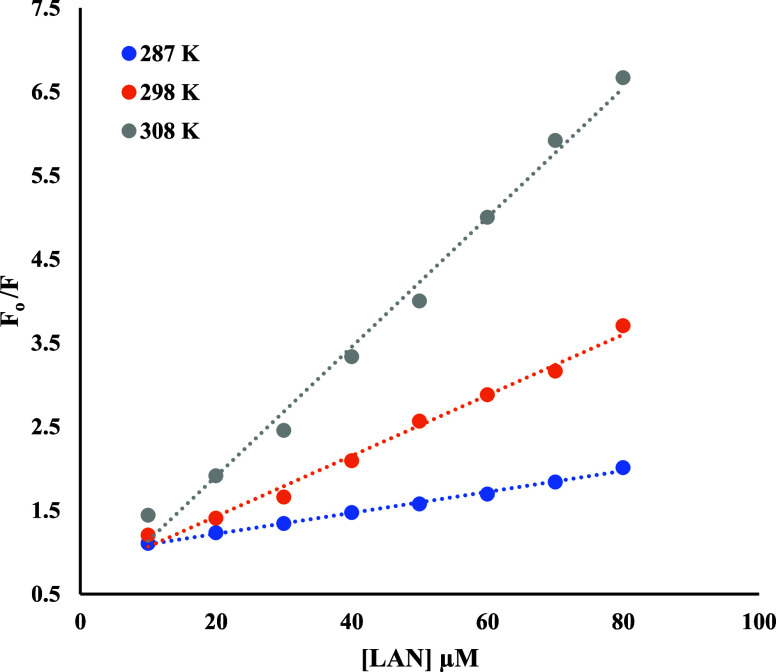
Stern–Volmer plots for LAN–BSA interaction, as derived from the fluorescence data shown in Figure 2.

**Table 1:** Binding Characteristics of LAN–BSA Interaction

*T* (K)	*K*_sv_ × 10^4^ (M^–1^)	*K*_q_ × 10^12^ (M^–1^ s^–1^)	*K*_a_ × 10^4^ (M^–1^)	Δ*S* (J mol^–1^ K^–1^)	Δ*H* (kJ mol^–1^)	Δ*G* (kJ mol^–1^)
287	1.25	1.25	1.25	+261.73	+52.63	–22.49
298	3.62	3.62	2.76			–25.38
307	7.73	7.73	5.24			–27.73

### 3.2 Binding Affinity and Thermodynamics of LAN–BSA Interaction

The LAN–BSA interaction’s *K*_a_ values were at 287, 298, and 307 K. The equation was used to convert fluorescence data from various temperatures into double logarithmic graphs

4 $$\log\left(F_{0}-F\right)/F=n\log K_{a}-n\log\left[1/\left(\left[L_{T}\right]-\left(F_{0}-F\right)\left[P_{T}\right]/F_{0}\right)\right]$$

Figure 4A illustrates the linear plots for the LAN–BSA interaction. Tabulated in Table 1 are the *K*_a_ values obtained from the y-intercepts of these graphs. The *K*_a_ values span from 1.25 to 5.24 × 10^4^ M^–1^, suggesting an intermediate strength of interaction between LAN and BSA. Based on these findings, it was concluded that BSA is capable of transporting LAN through the circulation and releasing it at the desired location.

**Figure 4:**
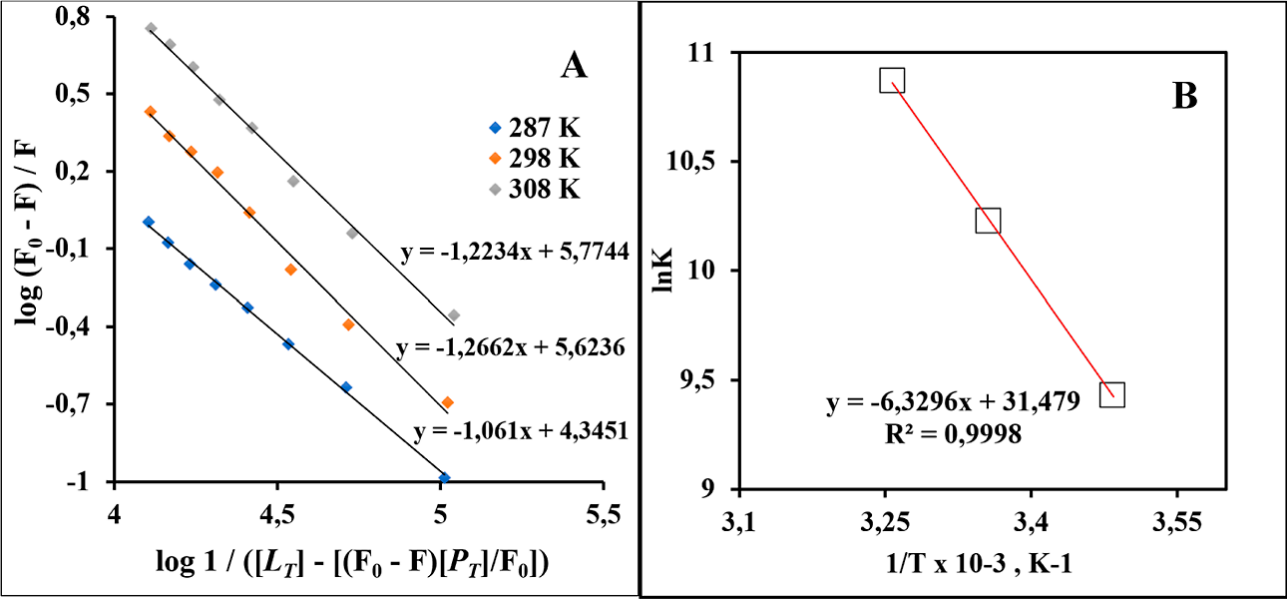
(A) Double logarithmic plots and (B) Van’t Hoff plot for LAN–BSA interaction.

Understanding how chemicals interact with proteins relies heavily on data related to thermodynamic characteristics. The primary forces that propel molecule–protein interactions include electrostatic, van der Waals, hydrophobic, and hydrogen (H) bonding.^66^ To find out which intermolecular interactions were involved in the creation of the LAN–BSA complex, researchers looked at thermodynamic characteristics such as changes in entropy (Δ*S*), enthalpy (Δ*H*), and free energy (Δ*G*). The Van’t Hoff plot (Figure 4B) was used to acquire the Δ*S* and Δ*H* values at three temperatures using eq 5, while eq 6 was used to secure the Δ*G* values.

5 $$\ln K_{a}=-\Delta H/RT+\Delta S/R$$

6 ΔG=ΔH−TΔS

According to Ross and Subramanian, if;

(i) Δ*H* > 0 and Δ*S* > 0, presence of hydrophobic interactions
(ii) Δ*H* < 0 and Δ*S* < 0, presence of van der Waals interactions and H-bonds,
(iii) Δ*H* < 0 and Δ*S* > 0, presence of electrostatic interactions are expected. However, electrostatic interactions are typically anticipated providing that the Δ*H* value is near to zero.^66^

In this context, the obtained Δ*H*, Δ*S*, and Δ*G* values are given in Table 1. The (+) Δ*H* value indicated that the LAN–BSA binding reaction was endothermic. Additionally, it was evident from the (−) Δ*G* values that LAN–BSA binding was spontaneous regardless of temperature. The great (+) Δ*H* value (+52.63 kJ mol^–1^) indicated hydrophobic interactions in the LAN–BSA complexation. Moreover, the (+) Δ*S* value (+261.73 J mol^–1^ K^–1^) stated that hydrophobic interactions were effective in the formation of the LAN–BSA complex formation. Therefore, according to experimental results, hydrophobic interactions were main force for LAN and BSA interaction due to both positive Δ*H* and Δ*S* values.

Moreover, Figure 5 shows the titration findings of BSA, PHN–BSA (1:1) and IBU–BSA (1:1) as a function of increasing LAN concentrations. The fluorescence quenching tendencies were seen to vary significantly more between the BSA and PHN–BSA combinations, but there was a lesser variation between the BSA and IBU–BSA mixtures (Figure 5). The results shown above clearly show that PHN provides less resistance to the LAN-induced decrease in the 347 nm fluorescence signal of BSA compared to IBU. These results indicate that the LAN molecule prefers to bind at the 1-site of BSA.

**Figure 5:**
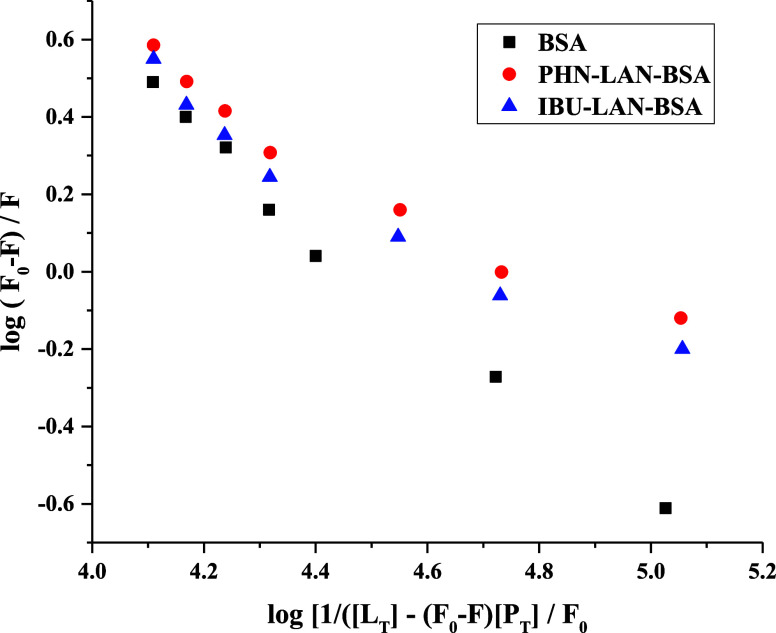
LAN-induced quenching of BSA fluorescence signals at 347 nm in the absence and presence of PHN and IBU. Conditions: [BSA] = [PHN] = [IBU] = 3 μM; [LAN] = (10–80) μM with 10 μM regular intervals; λex = 280 nm; buffer = PBS 7.4; *T* = 298 K.

### 3.3 3-D Fluorescence Spectral Results of the LAN–BSA Interaction

The 3-D fluorescence spectra were monitored to view the changes that would occur in the molecular environment near Trp and Tyr residues as a result of the addition of ligands to proteins. Fluorescence signals and contour maps of BSA and LAN–BSA mixtures ([LAN]/[BSA] = 40:3) are exhibited in Figure 6A,B. The positions of peaks and fluorescence intensities are also summarized in Table 2. In Figure 6A, peaks shown as “a” and “b” arise from Rayleigh scattering, and as Lakowicz stated, these peaks frequently come across in the proteins’ 3-D fluorescence signals.^61^ The peak located at λ_ex_ = 280 nm, designated “1” and the other peak located at λ_ex_ = 230 nm, designated “2” resulted from the fluorescence signals of Trp and Tyr residues.^18^ As seen in Figure 6B, the intensity of peaks “1” and “2” decreased significantly with the addition of LAN, whereas the peak “1” was not observed in LAN–BSA interaction solution (Figure 6B). Also, peak “2”, “a”, and “b” were seen for LAN–BSA interaction solution; however, a significant decrease was found when compared to the peak intensities observed in only BSA solution.

**Figure 6:**
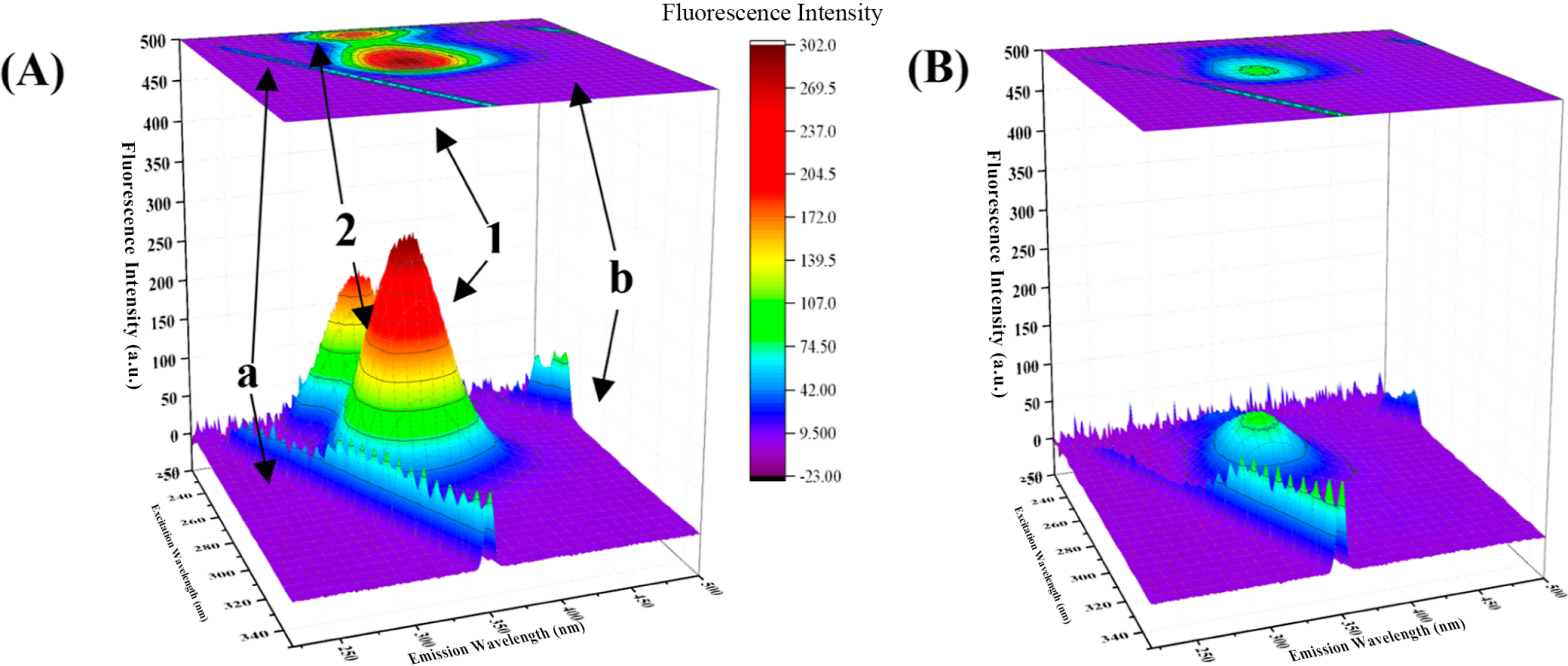
3-D fluorescence signals and contour maps of (A) 3 μM BSA and (B) 40:3­[LAN]/[BSA] mixture.

**Table 2:** Peak Positions and Intensity Values of 3-D Fluorescence Signals of BSA, LAN–BSA Mixtures, and LAN

system	peak	peak position [λ_ex_/λ_em_ (nm/nm)]	intensity
BSA (3 μM)	A	230/230 → 350/350	4.50 → 100.90
	B	250/500	74.52
	1	280/350	248.0
	2	230/343	198.0
[LAN]/[BSA] = 40:3	A	230/230 → 350/350	1.98 → 104.43
	B	250/500	34.78
	1	280/337	77.65
	2	230/332	3.44

When the results obtained from the 3-D fluorescence spectrum of the protein without and with 40 μM LAN were compared, it was found that the intensity values of peak “1” were reduced by 43% and the emission maxima was blue-shifted by 10 nm. Similarly, it was determined that the decline in the intensity values of peak “2” was 47% and the blue-shift in the emission maxima was 14 nm. The alterations in the fluorescence characteristics of these two peaks became more significant with the addition of more LAN (Table 2). Fluctuations in the spectral features of peaks 1 and 2 with the addition of LAN clearly indicated that the molecular environment around the protein fluorophores changed due to complex formation between LAN and BSA.

### 3.4 Synchronous Fluorescence Spectral Results of the LAN–BSA Interaction

Synchronous fluorescence spectroscopy can be helpful in the investigation of albumin-drug interactions.^67^ It offers comprehensive insights into conformational alterations, quenching methods, binding locations, and thermodynamic characteristics, consequently augmenting the comprehension of drug binding and its impact on albumin structure and function.^66^ Synchronized fluorescence spectra explain polarity changes at Try residues when Δλ is 15 nm, while they explain polarity changes at Trp residues when Δλ is 60 nm.^68^ As the concentrations of LANs rise, Figure 7A,B illustrate the variations in the synchronous fluorescence signal at Δλ of 15 and 60 nm for BSA. In both instances, the BSA intensity decreased with LAN. Nonetheless, it was observed that the decline in the fluorescence signal of the Trp residue was significantly greater than the decrease in the fluorescence signal of the Try residue. These outcomes hinted that the LAN–BSA interaction significantly affected polarity and hydrophobicity in the microenvironment near Trp residues, and the binding site was close to Trp residues while having a low impact on the microenvironment near Try residues. Additionally, LAN-triggered changes in BSA fluorescence signals at 15 and 60 nm are shown in Figure 7C. Quantitatively, the addition of LAN caused an approximately 78.42% decrease in the intensity values of BSA at Δλ = 15 nm, while it caused an approximately 87.25% decrease at Δλ = 60 nm (Figure 7C). This clearly suggests that LAN binds closer to Trp than Try residue. In addition, a 4 nm blue shift was clearly found in the λ_max_ value of the Trp residue, while no change was observed in the λ_max_ value of the Try residue. The blue shift in the emission λ_max_ value indicates that the LAN association with BSA altered the microenvironment near the Trp residue. These findings support data obtained from 3-D fluorescence spectra indicating that LAN binding to BSA leads to microenvironmental changes near the Trp residue.

**Figure 7:**
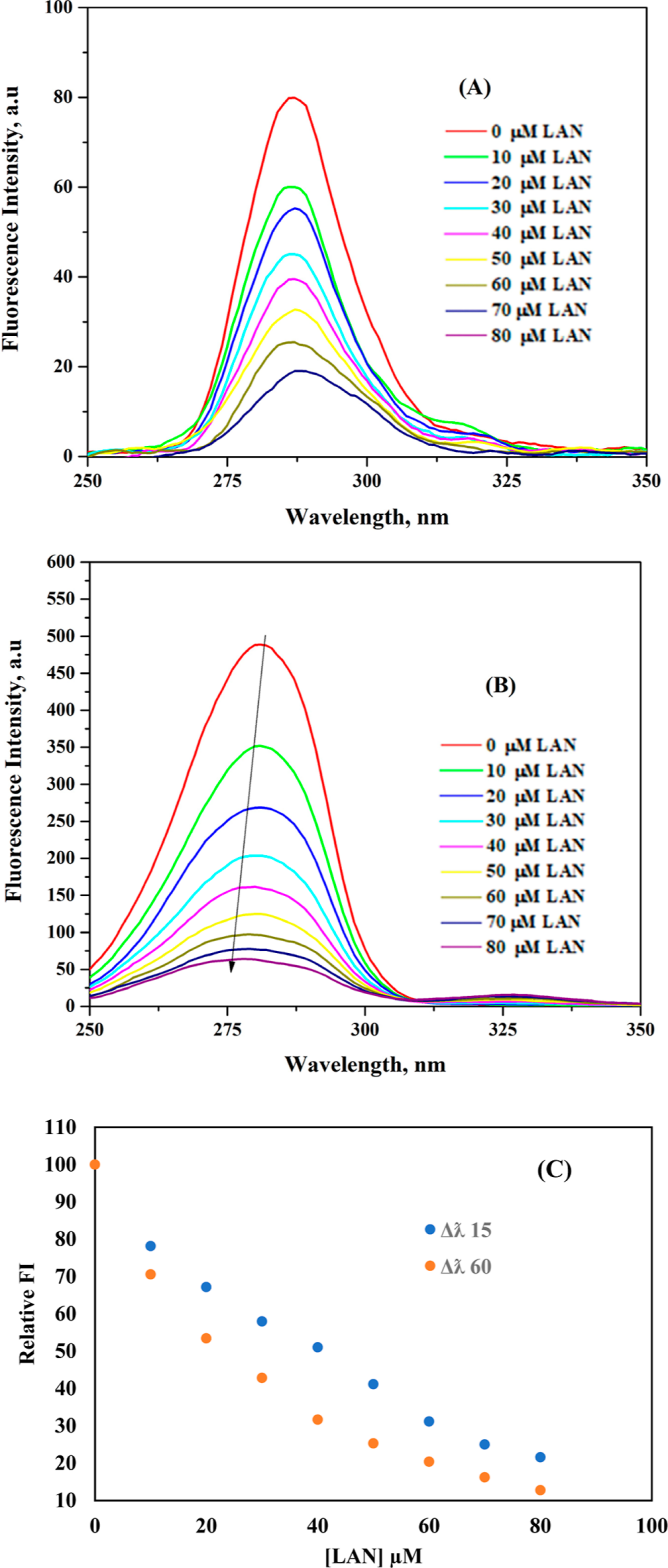
Synchronous fluorescence signals of 3 μM BSA upon rising (top to bottom) LAN concentrations (0–80 μM with 10 μM intervals), as registered using Δλ of (A) 15 nm and (B) 60 nm. (C) Plot displaying the relative FI of BSA upon increasing [LAN]/[BSA] molar ratios.

### 3.5 Electrochemical Investigation of LAN–BSA

Electrochemical techniques are widely used to study the interactions between biological macromolecules and small-molecule ligands, providing valuable insights into the binding mechanisms and conformational changes. Since BSA is a nonelectroactive molecule, its interaction with an electroactive ligand can only be monitored by changes in the electrochemical response of the ligand in solution. If a ligand binds to BSA on the electrode surface, the electron transfer process of the electroactive probe is hindered, leading to a reduction in peak current.^44,68^

In this study, the interaction between lansoprazole (LAN) and BSA was investigated using DPV. The concentration of LAN was kept constant at 50 μM, while the BSA concentration was systematically varied between 2 and 12 μM. Each solution mixture (3 mL) was incubated at room temperature for 30 min before electrochemical measurements. The DP voltammograms (Figure 8) were recorded in phosphate buffer solution (PBS, pH 7.4) at a bare GCE. A notable reduction in the anodic peak of LAN was seen when the content of BSA increased in a solution containing 10 μM LAN at physiological pH. Furthermore, in the absence of BSA, the peak potential of LAN was 0.95 V; nevertheless, with the addition of BSA at its maximum concentration, the LAN signal potential increased to 1.08 V, indicating a significant positive shift in the LAN signal potential. This shift suggests alterations in the microenvironment of the LAN, likely due to structural changes in the surrounding medium during BSA binding. The observed decline in peak currents indicates that the free concentration of LAN in solution decreased as a result of its interaction with BSA, leading to the formation of an electrochemically inactive complex. These findings confirm the binding interaction between LAN and BSA, highlighting the potential structural rearrangements in BSA upon ligand binding.

**Figure 8:**
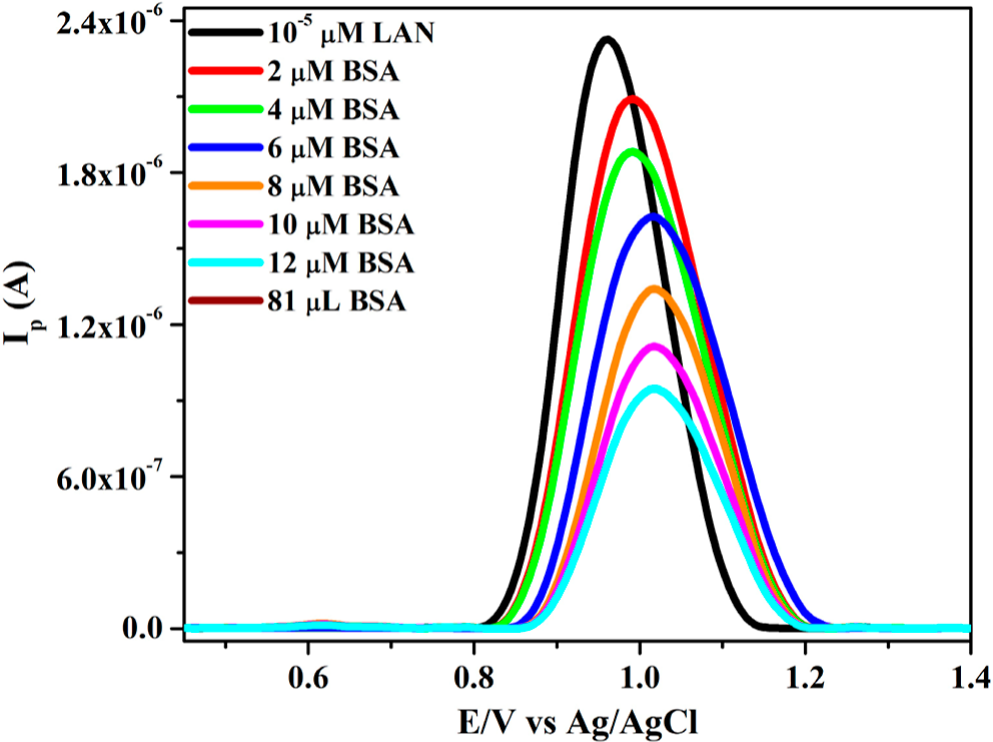
Electrochemical signals of 10 μM LAN upon rising (top to bottom) BSA concentrations (0–12 μM with 2 μM intervals).

### 3.6 Circular Dichroism (CD) Results

CD spectroscopy was employed to investigate the secondary structural alterations of BSA upon binding with the ligand LAN at a 1:1 molar ratio (3 μM BSA: 3 μM LAN). The CD spectrum of native BSA displayed characteristic negative peaks at 208 and 222 nm, which are hallmark features of α-helical structures. The ellipticity exhibited a progressive increase in negativity from 250 nm, peaking at approximately 221 nm with an MRE value of −20,159 deg·cm^2^·dmol^–1^, before gradually becoming less negative. This spectral profile aligns well with the expected CD signature of proteins rich in alpha-helices. Upon ligand binding, the BSA/LAN complex showed a marked reduction in the intensity of the negative peaks at 208 and 222 nm, indicating a significant conformational change in the protein structure. The α-helical content, calculated using the mean residue ellipticity (MRE) at 208 nm, was determined to be 62.7% for pure BSA and 11.8% for the BSA/LAN complex. A prominent negative peak observed at approximately 217 nm in the BSA/LAN spectrum suggests a potential structural transition, possibly toward beta-sheet or disordered conformations, as beta-sheets typically exhibit minima in the 215–217 nm range. Further supporting this structural rearrangement, the ellipticity ratio of the 208 to 222 nm peaks decreased, and a slight red shift in the position of the negative peak minimum was noted, both of which are indicative of partial unfolding or conformational changes induced by the ligand. The analysis reveals a substantial reduction in α-helical content from 62.7% in native BSA to 11.8% in the BSA/LAN complex, underscoring the disruptive effect of ligand binding on the protein’s helical framework. This significant decrease suggests that the interaction with LAN likely promotes the formation of beta-sheet structures or increases the proportion of disordered regions within the protein. Such conformational shifts are consistent with the known effects of ligand binding, which can lead to structural destabilization or partial unfolding, particularly impacting α-helical regions. These findings highlight the utility of CD spectroscopy as a sensitive technique for studying ligand-induced conformational dynamics in protein–ligand interactions.

### 3.7 FTIR Results

FTIR spectroscopy was employed to further investigate the structural changes in BSA induced by the LAN interaction. Figure 10 presents the FTIR spectra of pure BSA (black line) and the BSA-LAN complex (red line). The comparative analysis reveals significant spectral changes upon LAN binding, confirming the formation of a drug–protein complex and alterations in BSA’s secondary structure. A broad and intense absorption band around 3300 cm^–1^, typically attributed to the stretching vibrations of –OH and –NH groups, was prominent in the native BSA spectrum.^69^ This band exhibited a noticeable shift and decrease in intensity upon LAN interaction, suggesting the involvement of hydrogen bonding between LAN and BSA functional groups, particularly in regions associated with polar side chains. Moreover, the amide I (∼1650 cm^–1^) and Amide II (∼1540 cm^–1^) bands, characteristic of the protein’s secondary structure (α-helix and β-sheet content),^69^ also indicated distinct changes in both position and intensity. These alterations were indicative of conformational modifications in BSA upon ligand binding. The Amide I band, arising from C=O stretching vibrations, slightly shifts and decreases in intensity, suggesting perturbations in α-helical content. Likewise, the Amide II band-associated with N–H bending and C–N stretching-undergoes intensity reduction, supporting the hypothesis of reduced helical structure and increased disorder or β-sheet content. Also, these findings strongly corroborate the CD spectroscopy results (Figure 9), which demonstrated a substantial decrease in α-helix content, from 62.7% in native BSA to 11.8% in the BSA/LAN complex. Both FTIR and CD spectra indicated that LAN binding induces partial unfolding and a transition toward less ordered secondary structures in BSA. The FTIR region between 1000 and 1200 cm^–1^, typically associated with C–O stretching and skeletal vibrations, also reveals altered transmittance patterns in the BSA-LAN complex, implying interactions involving LAN’s aromatic or polar groups. Therefore, these spectral shifts confirm that LAN binding leads to significant structural rearrangements in BSA, including hydrogen bonding and hydrophobic interactions that disrupt the native α-helical framework and potentially promote β-sheet or random coil conformations. The parallel interpretation of FTIR and CD results provides robust evidence of the conformational sensitivity of BSA upon LAN binding and strengthens the structural conclusions of this study.

**Figure 9:**
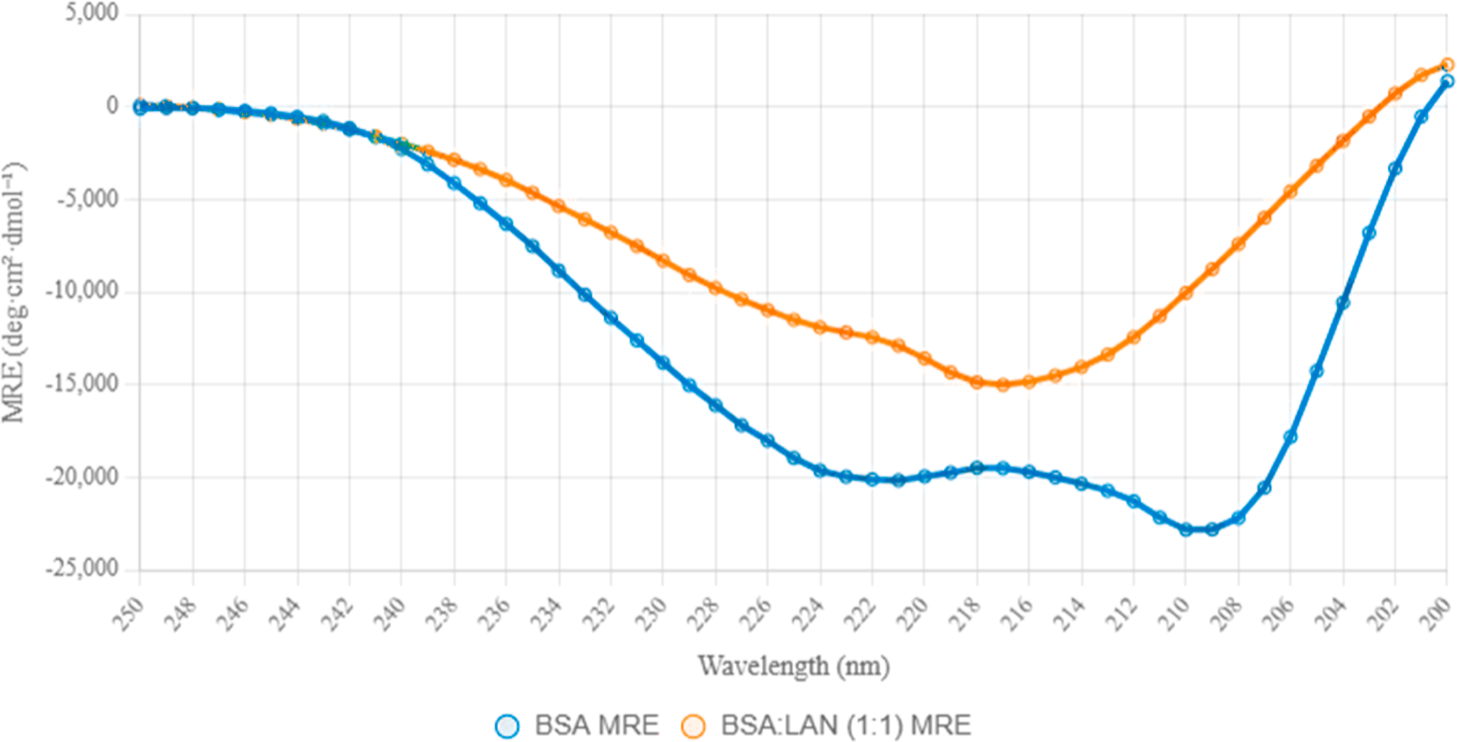
CD spectra of the protein and LAN/protein (1:1) mixture, obtained in the far-UV.

**Figure 10:**
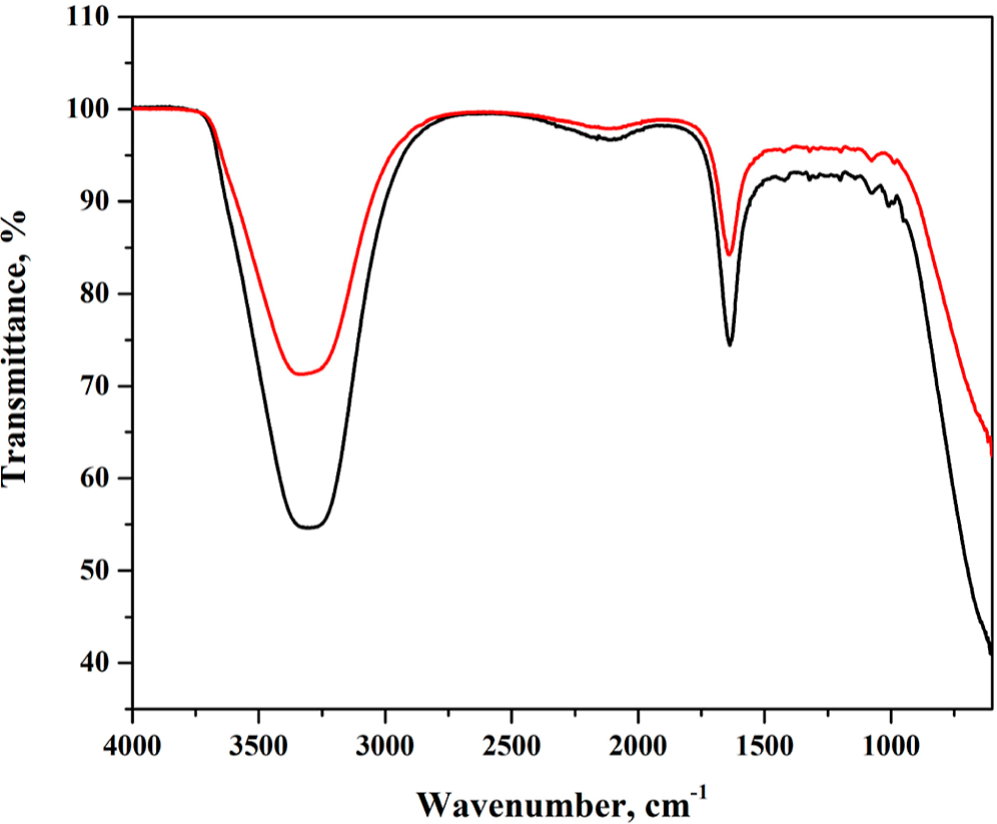
FTIR spectrum result of only BSA solution (black) and LAN/BSA; 4:1 (red).

### 3.8 Molecular Docking Results of LAN–BSA Complexes

Molecular docking experiments were designed to investigate the molecular relationships between the binding regions of LAN and BSA to validate our findings regarding competing site-marker displacement. Analysis of computational docking discloses the atomic-level insights by imagining and anticipating the relationship of a ligand–protein system (Anitha et al., 2022).^70^ The optimal binding mechanism of LAN to the BSA binding site was revealed through a specific position network at two distinct protein domain locations, which are allocated as Sites I and II. In the 100 docking experiments conducted on LAN–BSA complexes, many individual conformational clusters were observed at sites I and II of BSA (Figure 11). The predominant clusters at the specific Sites exhibited the following conformations and average binding energy: 19 (−37.03 kJ mol^–1^) at Site I, and 17 (−26.82 kJ mol^–1^) at Site II. Such outcomes seem to imply that generated clusters at Site I exhibited a notably lower average binding energy in opposition to the obtained clusters at Site II. Furthermore, the binding values of the lowest calculated energy for Site I were clarified to be (−38.28 kJ mol^–1^) while for Site II (−30.02 kJ mol^–1^). In contrast to Site II, the obtained energies of the lowest binding and the average binding (Figure 11) at Site I greatly forecast the first selection of LAN for Site I of BSA. The proposed LAN binding energy for Sites I and II of BSA is exemplified in Figure 12.

**Figure 11:**
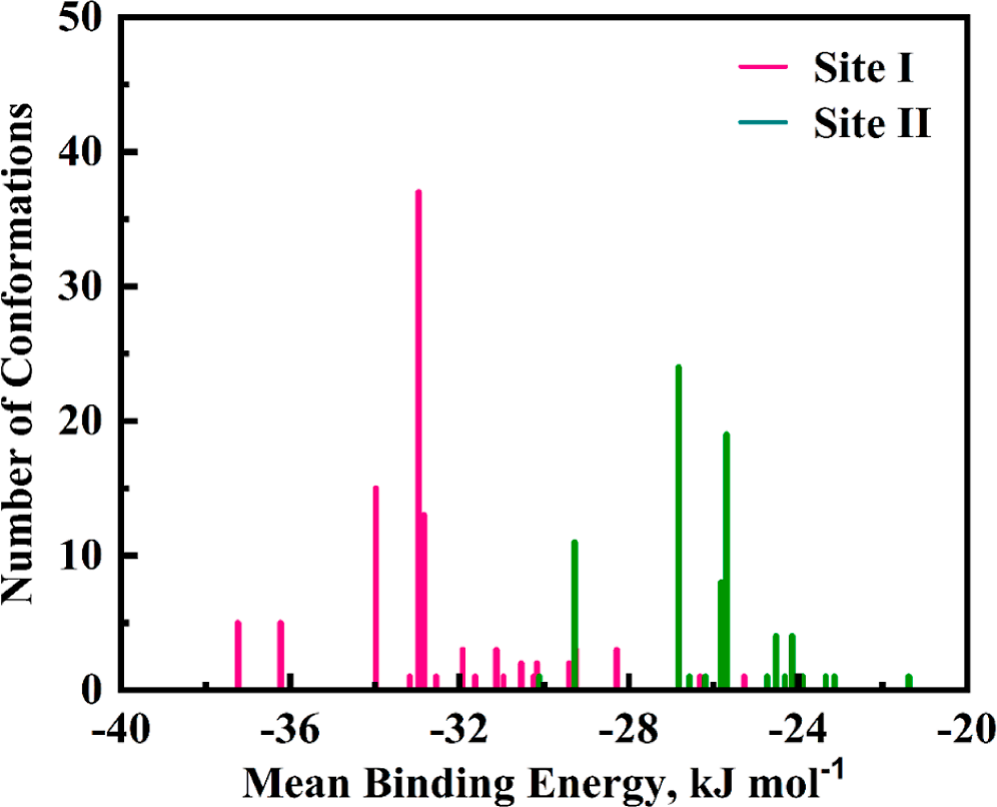
Analysis of 100 molecular docking simulations reveals clusters with the highest and lowest densities for LAN–BSA associations at Sites I and II, respectively.

**Figure 12:**
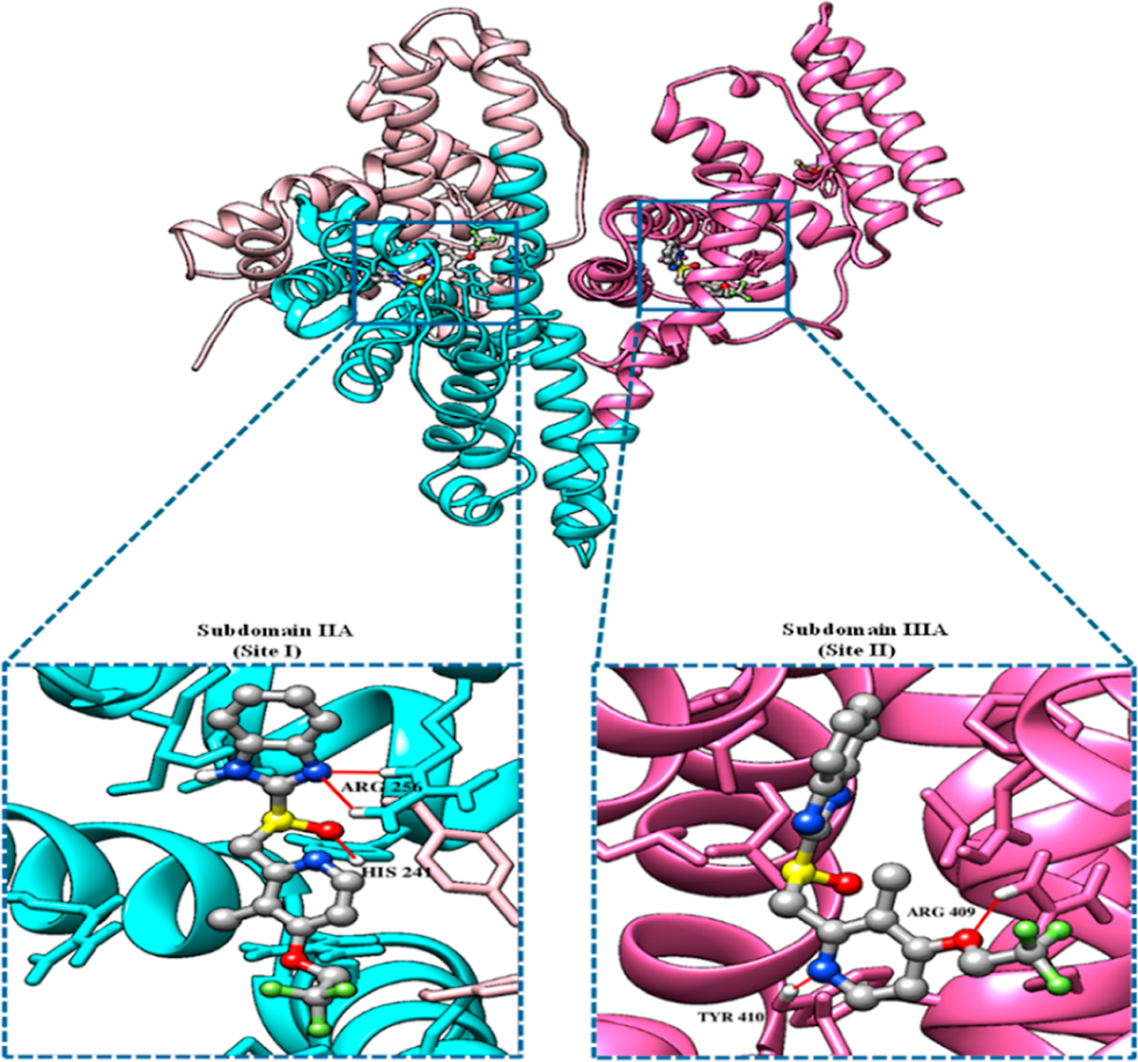
Illustrates the projected binding position of the binding energy arrangement of LAN in Sites I and II of BSA, respectively. The three domains within BSA are represented by the pink, cyan, and hot pink colors. Magnified images demonstrate the formation of H-bonds (red) in the LAN–BSA complexes.

The assessment of LAN binding of LigPlot+ at various binding areas of BSA revealed the development of hydrogen bonds concerning the LAN and residues of BSA along with the residues of the surrounding molecules (Figure 13A,B). The LigPlot+ evaluation (Figure 13A) indicated that most of the surrounding residues, including Ser 286, Ala 260, Glu 152, Ser 191, Ala 290, Ile 263, Arg 217, Arg 198, Leu 237, and Ile 289, were nonpolar, indicating that hydrophobic interactions among LAN and BSA were more dominant at Site I, the favored position for LAN binding. The establishment of three hydrogen bonds among LAN and residues, (2) Arg 256 and His 241, permitted the stability of the complex of LAN–BSA at Site I of BSA (Figure 13A and Table 3). Moreover, Site II also displayed the emergence of two hydrogen bonds (Figure 13B; Table 3). Furthermore, based on the previous findings, the complex produced by LAN to the BSA is more constant at Site I since it generates more hydrogen bonds and displays more hydrophobic interactions as well as having a lower binding energy (more negative) than at Site II. Thus, LAN is possibly more expected to attach to BSA at Site I.

**Figure 13:**
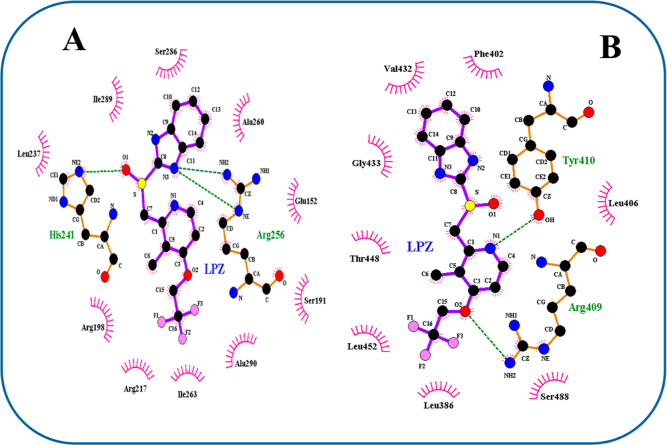
Results of 100 docking experiments show the emergence of H-bonds and hydrophobic interactions between BSA amino acid residues and the LAN molecule. (A) LAN–BSA interaction at Site I of BSA and (B) LAN–BSA interaction at Site II of BSA.

**Table 3:**
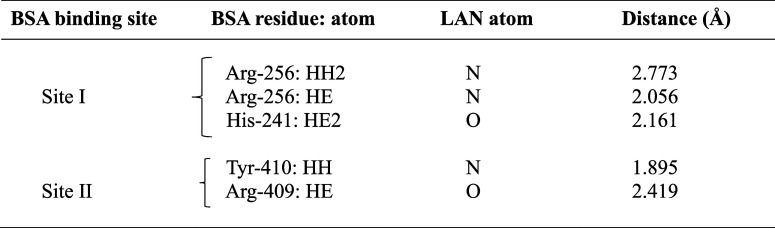
Docking Study Predicted Hydrogen Bonding between BSA and LAN at Sites I and II

### 3.9 Evaluation of MD Simulations of LAN–BSA System

The chemical and structural stability of the LAN–BSA mixture was investigated by employing MD simulations.^54^ The study was focused on identifying the reliability and relationships of the LAN and BSA frameworks at site I by tracking the trajectory produced during the 100 ns simulation run. Figure 14A shows the dependability of methods shown by a median total energy of 1.456 × 10^6^ kJ mol^–1^, a standard temperature of 300.004 K, and a pressure of 1.075 bar, respectively. The LAN maintained its association with Site I of BSA over the 100 ns simulation run. LAN remained inside the binding area of BSA Site I, as evidenced by the generated distance among the complexes of LAN and BSA (Figure 14B) during the simulation session at a value of 0.2 nm. The compactness of the BSA structure was anticipated to remain consistent throughout the simulation, as evidenced by the (*R*_g_) radius of gyration.^70^ In the LAN-bound form, the BSA structure remained uniformly compact over 100 ns, as evidenced by a *R*_g_ value of 2.70 nm (Figure 14C). Moreover, the RMSF value (root-mean-square fluctuation) indicated that many of the residues in the protein, which are amino acids, displayed typical resilience (<0.30 nm) upon LAN interaction except for distinct regions: residues 116–119, 182–185, 501–507, and 560–563 (Figure 14D). These undulations may be attributed to the remarkable adaptability of spiral formations, which are typically acknowledged for their attraction to these residues.^71^

**Figure 14:**
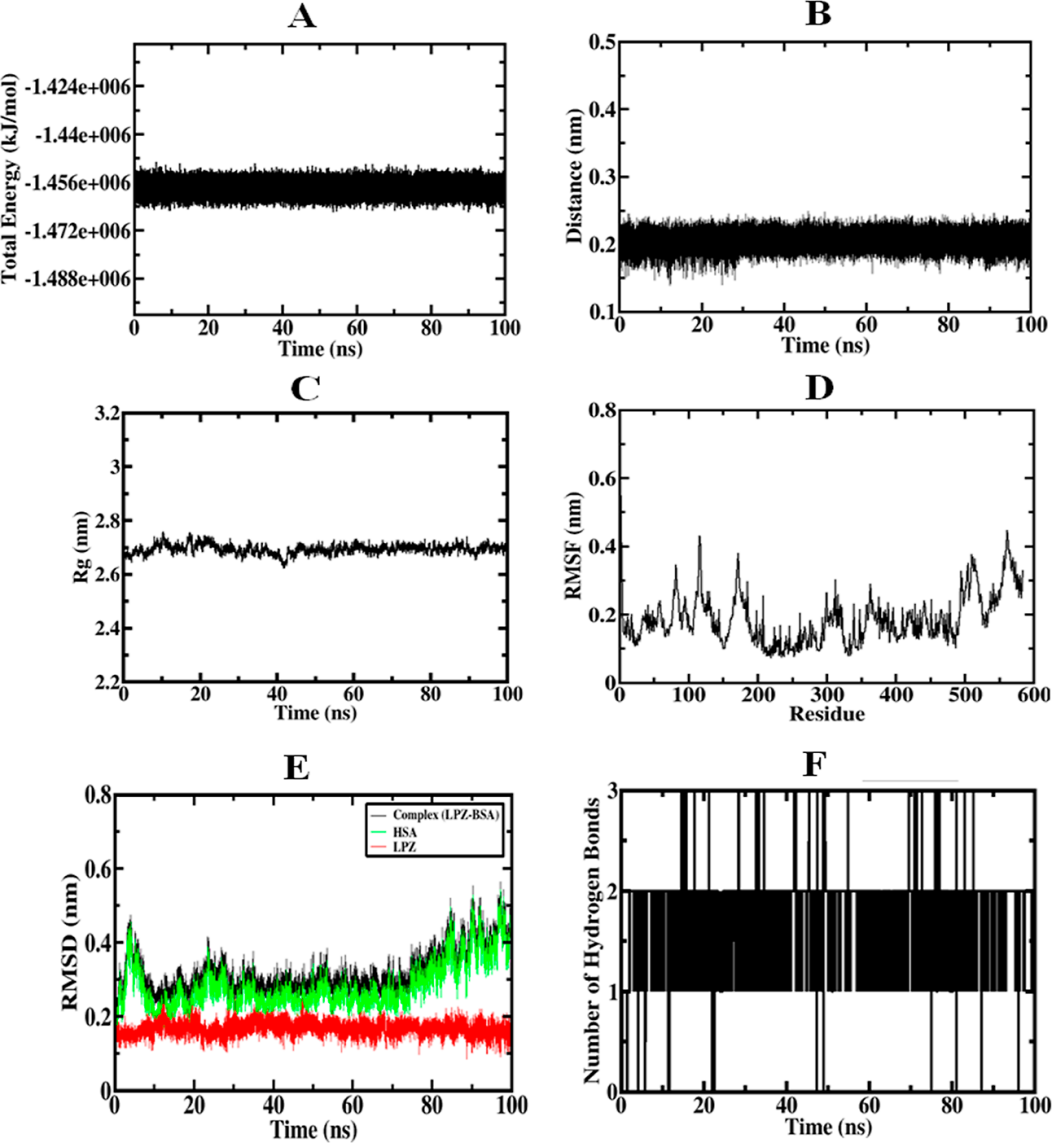
Graphs displayed by XMgrace to analyze the dynamics model of the system over simulation runs of 100 ns at Site I of BSA. (A) Total energy, (B) distance between LAN and BSA, (C) *R*_g_ for BSA, (D) RMSF for BSA, and (E) rmsd of the LAN, BSA, and LAN–BSA complex, and (F) creation of the H-bonds within LAN and BSA.

The root-mean-square deviation (rmsd) of the produced trajectories was analyzed to the initial complex setup during the simulation to provide a comprehensive assessment of the technique’s constancy and individual configuration. The generated rmsd plot (Figure 14E) generally demonstrated the complex stability of the LAN–BSA structure, BSA, and LAN across a period of 100 ns. A stable complex emerged, as indicated by a standard rmsd value of 0.31 nm. The evaluation of the stability between BSA and LAN was further explored throughout the simulation, particularly on the generated hydrogen bonds. The simulation repeatedly developed H-bonds between BSA and LAN, as evidenced by the potential for the formation of three H-bonds (Figure 14F). It suggested that the LAN binding capacity on BSA at Site I was enhanced by such H-bonds. This docking finding was supported by all MD simulation evaluations, indicating the stability of LAN to bond with Site I of BSA.

## 4 Conclusions

In conclusion, this study provides an integrated spectroscopic, electrochemical, and computational investigation of the interaction between LAN and BSA. Fluorescence quenching analyses, supported by UV–vis absorption and circular dichroism spectroscopy, revealed that LAN binds to BSA through a spontaneous and predominantly hydrophobic mechanism, inducing slight conformational rearrangements in the protein microenvironment. The electrochemical results further confirmed the interaction by demonstrating measurable changes in the oxidation peak potential and current intensity, consistent with static quenching behavior.

Molecular docking and dynamics simulations corroborated the experimental findings, identifying the principal binding site within Sudlow site I of BSA and highlighting stabilizing hydrophobic and van der Waals forces. The good agreement between in silico and in vitro data confirms the reliability of the proposed interaction mechanism.

As a summary, the present work enhances the understanding of proton pump inhibitor–albumin interactions and contributes to the prediction of the pharmacokinetic behavior of lansoprazole in biological systems. These insights are expected to guide future research on drug–protein binding mechanisms and to support the rational design of albumin-based drug delivery systems and safer pharmaceutical formulations.

## Data Availability

The data supporting this study are available within the manuscript.
